# The Other-Race-Effect on Audiovisual Speech Integration in Infants: A NIRS Study

**DOI:** 10.3389/fpsyg.2020.00971

**Published:** 2020-05-15

**Authors:** Yuta Ujiie, So Kanazawa, Masami K. Yamaguchi

**Affiliations:** ^1^Graduate School of Psychology, Chukyo University, Aichi, Japan; ^2^Research and Development Initiative, Chuo University, Tokyo, Japan; ^3^Japan Society for the Promotion of Science, Tokyo, Japan; ^4^Department of Psychology, Japan Women’s University, Kawasaki, Japan; ^5^Department of Psychology, Chuo University, Tokyo, Japan

**Keywords:** the McGurk effect, other-race effect, fNIRS (functional near infrared spectroscopy), infant, cross modal

## Abstract

Previous studies have revealed perceptual narrowing for the own-race-face in face discrimination, but this phenomenon is poorly understood in face and voice integration. We focused on infants’ brain responses to the McGurk effect to examine whether the other-race effect occurs in the activation patterns. In Experiment 1, we conducted fNIRS measurements to find the presence of a mapping of the McGurk effect in Japanese 8- to 9-month-old infants and to examine the difference between the activation patterns in response to own-race-face and other-race-face stimuli. We used two race-face conditions, own-race-face (East Asian) and other-race-face (Caucasian), each of which contained audiovisual-matched and McGurk-type stimuli. While the infants (*N* = 34) were observing each speech stimulus for each race, we measured cerebral hemoglobin concentrations in bilateral temporal brain regions. The results showed that in the own-race-face condition, audiovisual-matched stimuli induced the activation of the left temporal region, and the McGurk stimuli induced the activation of the bilateral temporal regions. No significant activations were found in the other-race-face condition. These results mean that the McGurk effect occurred only in the own-race-face condition. In Experiment 2, we used a familiarization/novelty preference procedure to confirm that the infants (*N* = 28) could perceive the McGurk effect in the own-race-face condition but not that of the other-race-face. The behavioral data supported the results of the fNIRS data, implying the presence of narrowing for the own-race face in the McGurk effect. These results suggest that narrowing of the McGurk effect may be involved in the development of relatively high-order processing, such as face-to-face communication with people surrounding the infant. We discuss the hypothesis that perceptual narrowing is a modality-general, pan-sensory process.

## Introduction

Humans’ perceptual systems develop to adapt to the surrounding environments. It has been found that during the development of infants, exposure to specific faces and languages influences their sensitivity to face or speech, which is called as the perceptual narrowing (Werker and Tees, 1983, 2002; Pascalis et al., 2002; Lewkowicz and Ghazanfar, 2006). For example, it has been shown that 6-month-old infants can discriminate individual human and monkey faces, but older infants aged 9 months can only discriminate individual human faces (Pascalis et al., 2002). Even within human faces, perceptual narrowing occurs such that 3-month-old infants can recognize both own- and other-race faces, but the ability to recognize other-race faces is diminished in infants older than 6 months, which is called as the other-race effect (Kelly et al., 2007, 2009). In speech perception, it also has been shown that English-learning infants aged 6–8 months can discriminate phonetic contrasts in their native language (English) as well as a non-native language (Hindi), but infants aged older than 10 months are not able to discriminate non-native phonetic contrasts that do not exist in their native language (Werker and Tees, 1983, 2002). Furthermore, a couple of studies reported the presence of narrowing in the perception of musical rhythms (Hannon and Trehub, 2005a, b). These studies demonstrated that 12-month-old infants show an adult-like, culture-specific response pattern to musical rhythms (Hannon and Trehub, 2005b) in contrast to the culture-general response that is evident at 6 months of age (Hannon and Trehub, 2005b). Infants’ perceptual sensitivity to faces, spoken languages, and even musical rhythms is broader in the early months of development and narrows gradually by the end of the first year.

The timing of emerging narrowing is shared in face perception and speech perception, although the interaction of speed of perceptual narrowing in both domains remains discussed. Recent studies have investigated the correlations between perceptual narrowing in the face and speech domains (e.g., Krasotkina et al., 2018; Xiao et al., 2018). These studies have suggested that the speed of the developmental trajectories of perceptual narrowing in the speech domain is not necessarily correlated with that in the face domain within infants older than 8 months. Whether the narrowing process is driven by modality-general mechanisms (e.g., Pascalis et al., 2002) or by modality-particular mechanisms (e.g., Krasotkina et al., 2018) remains unclear.

Some studies have suggested that experiences play roles in the development of multisensory perception, especially in audiovisual speech perception (Lewkowicz and Ghazanfar, 2006; Pascalis et al., 2014). This implies that perceptual narrowing is a modality-general, pan-sensory process. That is, the basic and broadly tuned abilities of audiovisual speech perception are present in the early months and are gradually tuned to match the environment around infants during the first year of life (Lewkowicz and Ghazanfar, 2009; Pascalis et al., 2014). Indeed, along with increased exposure to native languages, an infant’s ability for audiovisual speech matching (Kuhl and Meltzoff, 1982; Patterson and Werker, 1999) develops to work limited to specific phenomes that are present in the native language, by 11 month-olds (Pons et al., 2009). In addition to language experience, Lewkowicz and Ghazanfar (2006) and Lewkowicz et al. (2008) demonstrated one aspect of the role of visual experience by measuring infants’ sensitivity to audiovisual associations for rhesus monkey vocalizations. They presented 4- to 10-month-old infants with two side-by-side rhesus monkey faces producing a coo call and grunt call in the presence of one of the corresponding auditory calls. In their results, 4-to-6-month-old infants preferred the face corresponding with the auditory call, but 8- and 10-month-old infants were not able to do that (Lewkowicz and Ghazanfar, 2006).

However, no prior study demonstrated the evidence for the role of experience with own-race-faces in the development of audiovisual speech perception. Here, we tested this issue in the context of McGurk effect (McGurk and MacDonald, 1976). The McGurk effect is a well-known illusion that demonstrates the influence of visual speech on voice perception (McGurk and MacDonald, 1976). An example of this illusion is when a movie of a mouth articulating the phoneme/ka/ is dubbed with a voice uttering a different phoneme, /pa/, observers tend to perceive an intermediate phoneme (/ta/). The McGurk effect is widely used as an index of the robustness of the influence of visual speech in adults (Sekiyama and Tohkura, 1991; Ujiie et al., 2018a) and children (Massaro et al., 1986; Sekiyama and Burnham, 2008). The McGurk effect has been observed from the preverbal stage of infant development (Rosenblum et al., 1997; Desjardins and Werker, 2004). By 4 months of age, infants can discriminate auditory syllables (e.g., Eimas et al., 1971; Jusczyk et al., 1978) and match an auditory voice with facial speech (e.g., Kuhl and Meltzoff, 1982, 1984; Patterson and Werker, 1999, 2002). At around 5 months of age, infants can integrate a voice with an incongruent facial speech and perceive the McGurk effect, regardless of syllable combination (Rosenblum et al., 1997; Desjardins and Werker, 2004). Rosenblum et al. (1997) habituated 5-month-old infants with the speech of auditory/va/ with visual /va/ and presented them with two test stimuli; auditory /ba/ with visual /va/, which causes the McGurk effect (/va/), and auditory /da/ with visual /va/, which is perceived as /da/. The results revealed that the infants showed dishabituation to the stimulus of auditory /da/ with visual /va/. Thus, the infants could integrate auditory /ba/ with visual /va/ and perceive the McGurk effect (/va/) like adults. Desjardins and Werker (2004) demonstrated the McGurk effect in infancy by using the stimulus of auditory /bi/ with visual /vi/, which causes the McGurk effect (/vi/) in adults.

This study shed light on the different brain responses to own-race and other-race faces in the McGurk effect. Previous studies have reported the different brain responses of face processing between own-race and other-race conditions (e.g., Balas et al., 2011; Timeo et al., 2019) and those of speech processing between native and non-native speech (e.g., Kuhl et al., 2014). However, those of the McGurk effect have not yet been reported. The neural basis of the McGurk effect has been investigated from infants (Kushnerenko et al., 2008) to adults (e.g., Beauchamp et al., 2010; Nath and Beauchamp, 2012). Several functional magnetic resonance imaging (fMRI) studies showed that the left superior temporal sulcus (STS), an area critical for the integration of auditory and visual speech information (Calvert et al., 2000), is responsible for the occurrence of the McGurk effect as well as the processing of audiovisual congruent syllables (in children, Nath et al., 2011; in adults, Nath and Beauchamp, 2012). In infants, Kushnerenko et al. (2008) found the neural basis of the McGurk effect by using event-related brain potentials (ERPs). Their results showed that the ERP responses to the McGurk-type stimulus (audio /ba/ with visual /ga/) were similar to that to the audiovisual-matched stimulus (audio /ba/ with visual /ba/) rather than to that of the audiovisual-mismatched stimulus (audio /ga/ with visual /ba/).

In this study, we used a functional brain activity imaging technique, functional near-infrared spectroscopy (fNIRS) to measure infant brain activities. This technique is reliable and valid for measuring brain activity in infants and is also easier to conduct in infants than fMRI. Previous studies from our research group have revealed that increased hemodynamic responses of temporal regions in infants’ brain in reaction to processing faces (Otsuka et al., 2007; Kobayashi et al., 2018), color (Yang et al., 2016), and audiovisual matching of material information (Ujiie et al., 2018b). Especially, it has been shown that the cerebral hemoglobin concentrations in bilateral temporal brain regions includes brain activities in the STS area (e.g., Otsuka et al., 2007; Ujiie et al., 2018b). Based on these studies, we considered that fNIRS is informative for investigating the question of how experiences with faces of different races affect infants’ development of audiovisual speech integration.

In summary, the present study focused on infants’ brain responses to the McGurk effect to examine whether the other-race effect occurs in the activation patterns. In Experiment 1, we conducted fNIRS measurements to find the presence of a mapping of the McGurk effect in Japanese 8- to 9-month-old infants and to examine the difference between the activation patterns of own-race-face and other-race-face stimuli. We hypothesized that the left temporal region would selectively activate in response to the McGurk speech of the own-race face and audiovisual-matched speech but not to those of the other-race face. To support the fNIRS data, we confirmed whether the infants could perceive the McGurk effect only in the own-race face and not in the other-race face by using a familiarization/novelty preference procedure (Experiment 2).

## Experiment 1

### Methods

#### Participants

All infants were full term at birth (37+ weeks) and were healthy at the time of the experiments. The participants were 34 healthy Japanese infants (17 infants for the own-race-face condition, and 17 infants for the other-race-face condition) aged 8–9 months old (25 girls and 9 boys; mean age = 246.5 days, range = 226–283 days), all of who grew up in Japan. An additional 12 infants were excluded because of an insufficient number of successful trials (fewer than three trials for each condition) due to fussiness motion artifacts. Ethical approval for this study was obtained from the local Ethical Committee. Written informed consent was obtained from the parents of the participants.

#### Stimuli

We assigned 17 infants to the own-race condition, and 17 infants to other-race condition. Then, we conducted measurements of brain activity in the infants using the ETG-4000 system (Hitachi Medical Systems, Tokyo, Japan), the reliability, benefit, and variability of which were validated in our previous studies (e.g., Yang et al., 2016; Ujiie et al., 2018b).

For own-race-face and other-face-race conditions, we used audiovisual speech stimuli that were created from recordings of two women’s utterances for three syllables (/pa/, /ta/, and /ka/). In order to reduce the possible difference in accents between English and Japanese speakers, we used infant-directed speech (IDS), which has been shown to be relatively similar, regardless of the language (e.g., Piazza et al., 2017). The speakers were two women, a Japanese East-Asian (22 years old) and an English Caucasian (23 years old), both of whom are monolingual speakers. The visual stimuli (800 pixels × 450 pixels) were recordings of the speakers’ faces, made using a digital video camera (GZ-EX370; JVC Kenwood, Yokohama, Japan). The voices (digitized at 48 kHz with a 16-bit quantization resolution) were recorded using a dynamic microphone (MD42; Sennheiser, Wedemark, Germany). The visual and auditory stimuli were combined to create two matched and two McGurk stimuli using Adobe Premiere Pro CS6 (Adobe Systems, San Jose, CA, United States). For the McGurk stimuli, we combined /pa/ voice with the facial movement for /ka/, by adjusting the onset of voice (/pa/) based on the onset of the original utterance (/ka/). The congruency of the stimuli was based on the speech sound. The McGurk stimuli consisted of a voiced /pa/ with an incongruent articulation /ka/. Pink noise was added to the voices (the signal-to-noise ratio was 0 dB) to induce perception of the McGurk effect (e.g., Sekiyama and Tohkura, 1991; Ujiie et al., 2015). Finally, we created matched stimuli (auditory /pa/ and visual /pa/) and McGurk stimuli (auditory /pa/ and visual /ka/) for two speakers of different races (East Asian and Caucasian).

#### Apparatus

A 21-inch color cathode ray tube display with a resolution of 1,024 pixels × 768 pixels was used to present the visual stimuli. The display was placed in front of the infant at a distance of 40 cm. A pinhole camera was set below the display to monitor the infant’s looking behavior. The audio stimuli were presented at a sound pressure level of approximately 60 dB through two loudspeakers placed on the left and right sides of the display.

**FIGURE 1:**
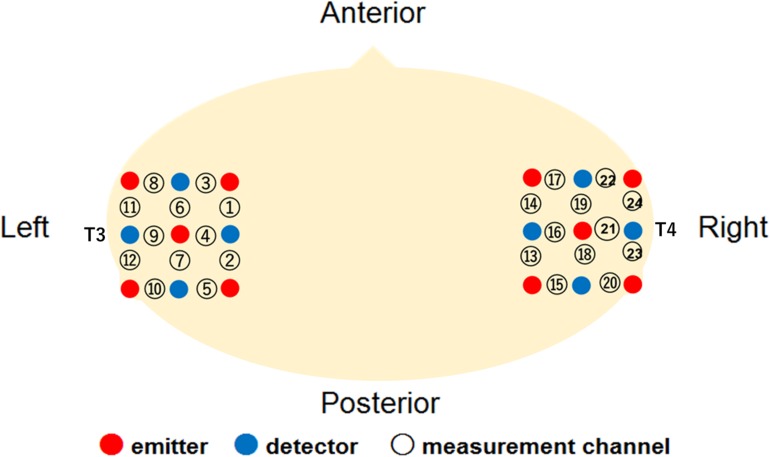
Location of the measurement channels in the current study.

The Hitachi ETG-4000 system (Hitachi Medical, Japan) was used to record the hemodynamic response simultaneously from 24 channels, with 12 channels for each right and left temporal area. The instrument generated two different wavelengths (695 and 830 nm) and measured the time course of changes in oxy-Hb, deoxy-Hb, and total-Hb with a 0.1-s time resolution. We used a pair of probes, each containing nine optical fibers (3 × 3 arrays) with five light emitters and four detectors. The optical fibers of each probe were kept in place with a soft silicon holder, and the inter-fiber distance was set at 2 cm. According to the International 10–20 EEG system, the center of each probe was placed at the T3 and T4 position for the measurement of the bilateral temporal regions (Figure 1). After positioning the probes, the experimenter checked whether the signals of the channels were appropriate to measure the hemodynamic responses via the ETG-4000 system, which automatically detects whether or not the probes were contacting the infant’s scalp correctly. The channels were rejected from the analysis if adequate contact between the fibers and scalp could not be achieved because of interference from hair.

#### Procedure

**FIGURE 2:**
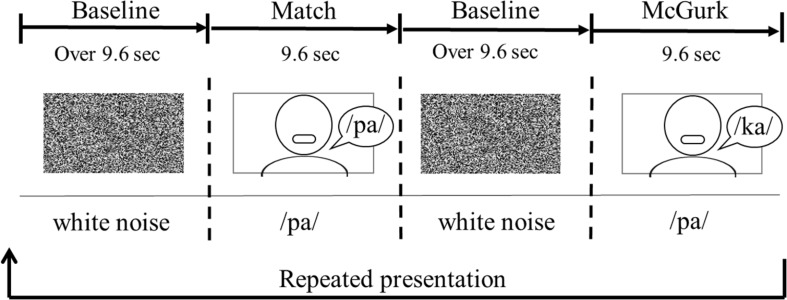
An example of the order of stimulus presentation.

Each infant was seated on her (or his) parent’s or an experimenter’s lap. The viewing distance was approximately 40 cm. The sequence of the stimulus presentation consisted of a baseline trial and two test trials (Figure 2). One test trial consisted of three presentations of match stimuli, and the other included three presentations of McGurk stimuli. The duration of the test trial was 9.6 s. Each test trial was presented alternately between the baseline trials. During the baseline trial, dynamic random dot patterns (800 pixels × 450 pixels) with an auditory white noise were displayed simultaneously once every 3.2 s. The baseline trial was controlled by the experimenter, and its duration was at least 9.6 s. The presentation order of the two test trials was randomly counterbalanced across infants. Each test trial was shown to the infants for a maximum of eight times.

The infants looked at the stimuli passively while their brain activity was recorded. They were allowed to look at the stimuli as long as they were willing to. Their behavior was recorded digitally throughout the experiment.

#### Data Analysis

According to the exclusion criteria of previous studies (e.g., Yang et al., 2016; Kobayashi et al., 2018), we removed trials from analysis if (1) the infants’ looking time in the test period was less than 60% of the total duration of the test period or if they became fussy, (2) the infant looked back to the experimenter’s or parent’s face during the preceding baseline period, or (3) motion artifacts were detected by the analysis of sharp changes in the time courses of the raw oxy-Hb data.

We used a Hitachi ETG system to convert the light intensity data of the two wavelengths for each Hb concentration. The values of oxy-Hb, deoxy-Hb, and total-Hb in each channel were calculated by using the difference of the intensities between wavelengths of light (695 and 830 nm) based on the modified Beer-Lambert law. After converting each Hb concentration, we checked for motion artifacts. In order to detect motion artifacts, we used the formula and criteria used in previous NIRS studies (e.g., Yang et al., 2016; Kobayashi et al., 2018). We first calculated the value by dividing the average raw data at four time points (mM × mm) by the average raw data at four time points thereafter. If the value was larger than 0.8, we defined that the data (trial) included a body movement artifact, and removed it from the analysis.

The raw Hb concentration changes from the individual channels were digitally band-pass-filtered at 0.02–1.0 Hz to remove longitudinal signal drift and noise from the instrument. We averaged the raw data of each channel across trials within each participant in a time series from 3 s before the test trial onset to 10 s after the test trial offset. From the time series of raw data of oxy-, deoxy-, and total-Hb, we calculated the *Z*-scores at each time point separately for the matched and mismatched conditions. The *Z*-scores, as the difference of the means between the baseline and test condition, were calculated using the following formula:

$$Z\,\mathrm{score}=\left(T\,e\,s\,t-M_{\text{baseline}}\right)/S$$

where *Test* represents the raw data values at each time point during test trials, For the value of *M*_baseline_, we used the mean of the raw data during the 3 s immediately before the beginning of each test trial. *S* indicates the standard deviation of the raw data during the same time period as *M*_baseline_.

### Results

**FIGURE 3:**
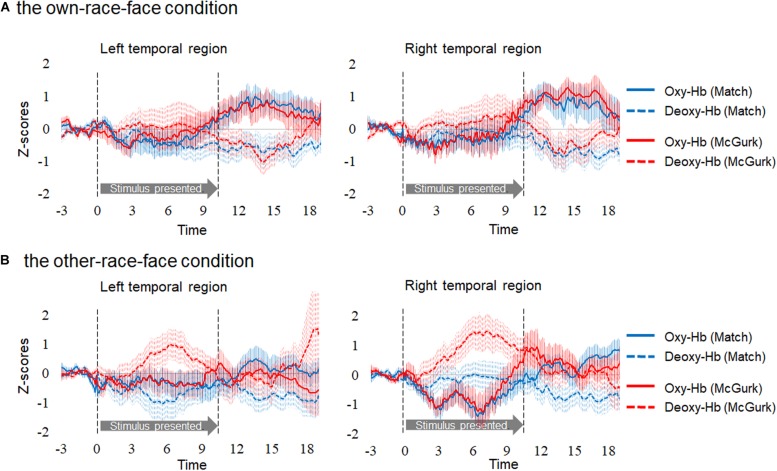
Time course of the changes in the oxygenated hemoglobin (Oxy-Hb) and deoxygenated hemoglobin (Deoxy-Hb) concentrations. Oxy-Hb and Deoxy-Hb concentrations were separately averaged in all groups during each condition in the left and right temporal regions. Panel **(A)** shows the results for the own-race-face condition, and **(B)** shows the results for the other-race-face condition. Solid lines represent the change in Oxy-Hb, and dotted lines represent the change in Deoxy-Hb. Blue lines and red lines represent the mean *Z*-score during the Match and McGurk trials, respectively. The vertical dashed lines at 0 and 9.6 s indicate the onset and offset of the test stimulus presentation, respectively.

Hemodynamic data were obtained from 34 infants, and included more than three valid trials for each test trial. On average, we obtained approximately five valid trials for each test trial in the two conditions. There were five valid trials (*SD* = 1.50, range: 3–7) for Match and five valid trials (*SD* = 1.00, range: 3–6) for McGurk in the own-race-face condition. There were 4.6 valid trials (*SD* = 1.17, range: 3–7) for Match and 4.7 valid trials (*SD* = 1.10, range: 3–6) for McGurk in the other-race-face condition. We normalized the raw data of the hemodynamic responses using the mean and standard deviation (SD) of the baseline period for each channel and each participant before applying statistical analyses, because the raw data could not be averaged directly between participants and channels. Subsequently, we averaged the *Z*-scores of the oxygenated hemoglobin (oxy-Hb) across the 12 channels in each hemisphere and compared them to the baseline. Figure 3 shows the time course of the average changes in concentration for oxy-Hb and deoxy-Hb during the presentation of the Match and McGurk trials for each race-face condition (results of total-Hb change are provided in the Supplementary Information). In the own-race-face condition, the oxy-Hb concentration in the left temporal region increased during both Match and McGurk trials. This increased activation reached a peak and started to return toward the baseline level between 12 and 16 s after stimulus onset. Such activation was not observed in the other-race-face condition.

In order to examine whether each temporal region was activated in response to audiovisual speech integration for each race-face, we conducted a two-tailed one sample *t*-test against zero response (baseline). As in common with infant studies of fNIRS (e.g., Issard and Gervain, 2017), we focused on concentrations of oxy-Hb. Firstly, to select the time window for averaged data, we compared oxy-Hb concentrations in each hemisphere in each condition against a baseline (*z* = 0) with cluster-based permutation tests. Such tests, which were successfully used in previous studies, can take into account temporal adjacency, clustering together samples that show a significant effect if they are adjacent in time (e.g., Maris and Oostenveld, 2007; Benavides-Varela and Gervain, 2017; Issard and Gervain, 2017). We first performed *t*-tests against baseline for each data point, then grouped data points temporally with a *t*-value greater than a standard threshold (*t* = 2), referred to in previous studies (e.g., Maris and Oostenveld, 2007; Benavides-Varela and Gervain, 2017). These analyses revealed a data-driven time window from 12 to 16 s after the stimulus onset.

**FIGURE 4:**
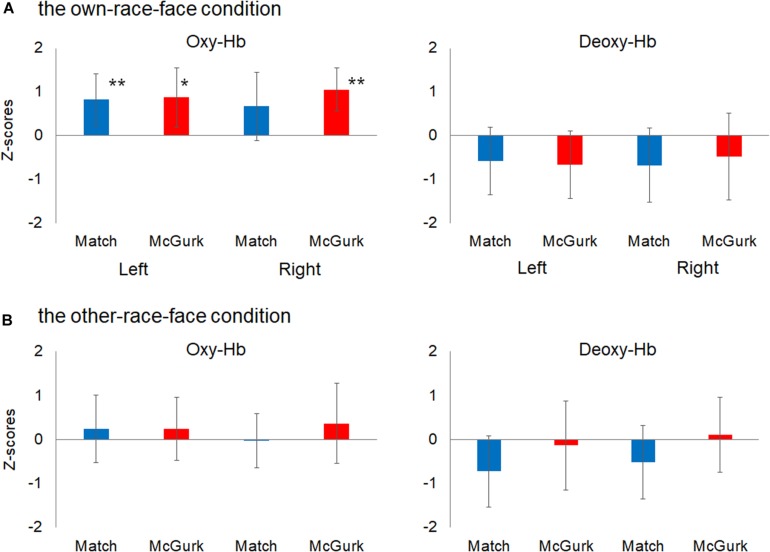
Mean *Z*-scores for oxygenated hemoglobin (Oxy-Hb) and deoxygenated hemoglobin (Deoxy-Hb) in all groups for the left temporal (left) and right temporal (right) regions. Panel **(A)** shows the results for Oxy-Hb (in the left panel) and Deoxy-Hb (in the right panel) for the own-race-face condition and **(B)** shows the results for the other-race-face condition. Each bar represents the mean *Z*-score for Oxy-Hb (or Deoxy-Hb) averaged across 12–16 s after stimulus onset. Blue bars and red bars represent the results for the Match and McGurk conditions, respectively. The error bars represent the 95% confidence interval of the mean. Asterisks indicate the significance level of the statistical differences against baseline (0): *p < 0.05, **p < 0.01.

We then performed statistical analyses with mean *Z*-scores during the 12–16 s after stimulus onset in the left and right temporal regions (Figure 4). A planned two-tailed one sample *t*-test with a zero response as the baseline was conducted for each region, with reference to previous studies of fNIRS in infants (e.g., Ujiie et al., 2018b). In the own-race-face condition, the concentration of oxy-Hb in the left temporal region increased significantly during both the Match [*t*(16) = 2.77, *p* = 0.028, false discovery rate (FDR) corrected, *d* = 0.92] and McGurk trials [*t*(16) = 2.55, *p* = 0.028, FDR corrected, *d* = 0.86]. In the right temporal region, the concentration of oxy-Hb increased significantly during the McGurk trials [*t*(16) = 4.55, *p* = 0.001, FDR corrected, *d* = 1.43] but not during the Match trials [*t*(16) = 1.41, *p* = 0.116, FDR corrected, *d* = 0.57]. In contrast, changes in the concentration of deoxy-Hb did not reach at a significant level in both temporal regions; the Match [*t*(16) = 1.56, *p* = 0.22, FDR corrected, *d* = 0.50] and McGurk trials [*t*(16) = 0.94, *p* = 0.46, FDR corrected, *d* = 0.57] in right temporal region, and the Match [*t*(16) = 1.46, *p* = 0.28, FDR corrected, *d* = 0.54] and McGurk trials [*t*(16) = 1.67, *p* = 0.36, FDR corrected, *d* = 0.33] in left temporal region. In the other-race-face condition, no significant activation for the concentration of oxy-Hb was found in the left temporal region during the Match [*t*(16) = 0.63, *p* = 0.99, FDR corrected, *d* = 0.21] and McGurk trials [*t*(16) = 0.65, *p* = 0.70, FDR corrected, *d* = 0.22], and in the right temporal region during the Match [*t*(16) = 0.11, *p* = 0.91, FDR corrected, *d* = 0.04] and McGurk trials [*t*(16) = 0.79, *p* = 0.99, FDR corrected, *d* = 0.27]. For the concentration of deoxy-Hb, no significant activation was found in the left temporal region during the Match [*t*(16) = 1.83, *p* = 0.35, FDR corrected, *d* = 0.61] and McGurk trials [*t*(16) = 0.26, *p* = 0.99, FDR corrected, *d* = 0.09], and in the right temporal region during the Match [*t*(16) = 1.23, *p* = 0.47, FDR corrected, *d* = 0.41] and McGurk trials [*t*(16) = 0.25, *p* = 0.99, FDR corrected, *d* = 0.09].

A further analysis was conducted to examine the cortical areas that potentially exhibit brain activity related to audiovisual speech integration. Based on the locations of the 10–20 cortical projection points, individual channels in the fNIRS measurement can be estimated to represent anatomical brain areas in infants’ brain (Lloyd-Fox et al., 2014). We then conducted one sample *t*-test against the zero response (baseline) on the *Z*-scores of oxy-Hb for each channel separately. The significantly activated channels are summarized in Table 1. In our setting of the channels’ location (Figure 1), the left superior temporal channel (ch 4) can be considered to reflect the activation of the left superior temporal region, according to the anatomical regions for the projection of each fNIRS channel in Lloyd-Fox et al. (2014). Focusing on the left superior temporal channel (ch 4), oxy-Hb increased significantly during both the Match [ch4; *t*(16) = 3.38, *p* = 0.046, FDR corrected, *d* = 1.11] and McGurk trials [ch 4; *t*(16) = 3.52, *p* = 0.034, FDR corrected, *d* = 1.13] in the own-race-face condition. In contrast, no significant activation was found in the other-race-face condition during the Match [ch 4; *t*(16) = 1.36, *p* = 0.29, FDR corrected, *d* = 0.27] or McGurk trials [ch 4; *t*(16) = 0.88, *p* = 0.52, FDR corrected, *d* = 22]. The responses from the channel 4 could be assumed to be associated with the activation of the left superior temporal area, which is related to the processing of audiovisual speech (e.g., Calvert et al., 2000; Nath and Beauchamp, 2012). To summarize, the individual channel analysis indicated significant differences in oxy-Hb responses in the left temporal regions between the two race-face conditions, which suggests that the left superior temporal area may be selectively activated in response to the audiovisual stimulus of the own-race face.

**TABLE 1:** Summary of significant channels from the individual channel analysis.

Comparison	Hemisphere	Significant channels	*t*-value	*p*-value (FDR corrected)	*d*
**The own-race-face condition**					
Match vs. baseline	Left	ch4	*t*(16) = 3.38	*p* = 0.046	*d* = 1.11
	Right	None	–	–	–
McGurk vs. baseline	Left	ch4	*t*(16) = 3.52	*p* = 0.034	*d* = 1.13
	Right	None	–	–	–
Match vs. baseline	Left/right	None	–	–	–
McGurk vs. baseline	Left/right	None	–	–	–

### Discussion

In Experiment 1, we conducted fNIRS measurements to find the presence of a mapping of the McGurk effect in Japanese 8- to 9-month-old infants and to examine the difference between the activation patterns of own-race-face and other-race-face stimuli. We conducted analysis for both oxy-Hb and deoxy-Hb, but obtained significant results only for oxy-Hb, which is common with infant studies (e.g., Issard and Gervain, 2017). Our results indicate that (1) the McGurk stimuli induced activations in the bilateral temporal regions, while the audiovisual-matched stimuli induced the activation in the left temporal region; and that (2) this activation pattern was found in the own-race-face condition but not in the other-race-face condition. These results would support our assumption that the infant brain activates in response to the McGurk effect when an own-race face stimulus is presented but not when an other-race face is presented.

We found a difference in the activation patterns between the audiovisual-matched and the McGurk stimuli in the own-race-face condition. The matched stimuli induced activation of the left temporal region, while the McGurk stimuli induced activation of the bilateral temporal regions. Our results suggest that the mapping of the McGurk effect in the infant brain was different from that found in adult studies (Beauchamp et al., 2010; Nath and Beauchamp, 2012). In adults, the left STS, which is important for processing audiovisual speech (e.g., Calvert et al., 2000; Beauchamp et al., 2004), is responsible for the McGurk effect (Beauchamp et al., 2010; Nath and Beauchamp, 2012). In infants, in addition to the left temporal region, the McGurk effect induces the activation of the right temporal region, which is important for processing faces (e.g., Otsuka et al., 2007; Kobayashi et al., 2018). The activation in the right temporal region may come from an infant’s need to process the speaking face of the McGurk stimuli, because the development of the McGurk effect is immature (e.g., McGurk and MacDonald, 1976; Sekiyama and Burnham, 2008).

To support our fNIRS data, we used a familiarization/novelty preference method (e.g., Yang et al., 2016; Sato et al., 2017) to confirm whether infants could perceive the McGurk effect in the own-race-face condition and not in the other-race-face condition. Similar to the fNIRS experiment, we used two race-face conditions, each of which consisted of two phases: the familiarization phase and test phase. In the familiarization phase, we presented infants with six familiarization trials, which repeated the McGurk stimulus (auditory /pa/ and visual /ka/) six times per trial. In the test phase, we presented the infants with the familiarized trials and a novel trial. The familiarized trial consisted of a repeated presentation of a voiced “/ta/” syllable and vegetable images six times. The novel trial consisted of repeated presentation of a voiced “/pa/” syllable with vegetable images six times. We expected that if infants can perceive the McGurk effect, they would become familiarized with the “/ta/” sound in the familiarization phase, thus they would look longer at the novel trial (/pa/) in the test phase. In our hypothesis, a significant preference for the novel trial in the test phase would result from the presence of audiovisual speech integration in the familiarization phase.

## Experiment 2

### Participants

Twenty-eight infants aged 8–9 months (15 girls and 13 boys; mean age = 254.6 days, range = 225–294 days), all of whom grew up in Japan, participated in the behavioral experiment (14 infants for the own-race-face condition, 14 infants for the other-race-face condition). The infants did not participate in Experiment 1. Another 8 infants were tested but were excluded from the analysis because of longer looking times in the last three trials than in the first three trials during the familiarization phase. Ethical approval for this study was obtained from the Ethical Committee at Chuo University. Written informed consent was obtained from the parents of the participants.

### Stimuli and Procedure

We used the McGurk stimulus and two auditory stimuli (/pa/ and /ta/), which were created from the same stimuli as used in Experiment 1. We set two conditions of speakers’ faces; the own-race-face (East Asian) and the other-race-face (Caucasian). The experiment task consisted of a familiarization phase and a test phase. The familiarization phase included six trials, and the test phase included two trials. In the familiarization phase, infants were familiarized to the sequence of repeated presentation of the McGurk stimulus (auditory /pa/ and visual /ka/) six times per trial. The test phase consisted of two trials; the familiarized and novel trials In these trials, we applied images of vegetables as non-face object stimuli to help infants focus more on the auditory syllable. In the familiarized trial, the voiced “/ta/” syllable was presented with images of vegetables six times. In the novel trial, the voiced “/pa/” syllable was also presented with images of vegetables six times. In each condition, the property of the speaker’s race and voice was constant. Each trial lasted 16.8 s and was preceded by the presentation of a fixation cue in the center of the monitor. The order of the presentation of two trials was randomly counterbalanced across infants. We conducted the experiments separately for each condition. Each infant was seated on their parent’s lap. The viewing distance was approximately 40 cm. The infants looked at the stimuli on the monitor without any active task. Their behavior was recorded digitally throughout the experiment.

The observer measured each infant’s looking time in offline video analysis. Blinded to the stimulus condition and the order of test trials, the observer recorded each infant’s looking time by pressing a key while the infant was looking at the display. When the infant looked away from the display, no recording was made. We removed data from the analysis if an infant’s looking times in the last three trials were longer than in the first three trials during the familiarization phase (e.g., Ujiie et al., 2018b). Inter-observer reliability was calculated based on the correlation between the looking times rated by both observers in all conditions. The Pearson correlation between the two observers’ ratios demonstrated that the rating reached a sufficiently reliable level (*r* = 0.91).

### Apparatus

A 21-inch color cathode ray tube display with a resolution of 1,024 pixels × 768 pixels was used to present the visual stimuli. The display was placed in front of the infant at a distance of 40 cm. A pinhole camera was set below the display to monitor the infant’s looking behavior. The audio stimuli were presented at a sound pressure level of approximately 60 dB through two loudspeakers placed on the left and right sides of the display.

### Results

#### Familiarization Trials

The mean total looking time across the first half and second half of familiarization trials in the own-race-face condition and the other-race-face condition are summarized in Table 2. To examine whether an infant’s fixation time during the familiarization trials differed between two race-face conditions, we conducted a mixed ANOVA with trials (the first half and second half of familiarization phase) as a within-participants factor and the stimulus conditions (the own-race-face and the other-race-face) as a between-participants factor. The ANOVA showed a significant main effect of trials [*F*(1,26) = 19.14, *p* < 0.01, η^2^ = 0.42]. A main effect of the stimulus [*F*(1,26) = 0.00, *p* = 0.99, η^2^ = 0.00] and an interaction [*F*(1,26) = 0.12, *p* = 0.73, η^2^ = 0.0004] were not significant. These results revealed that all infants became familiarized to the McGurk stimuli without any differences in fixation times during the familiarization phase between the own-race-face condition and the other-race-face condition.

**TABLE 2:** Mean total looking times (s) across the first half and second half of familiarization trials in both the own-race-face condition and the other-race-face condition.

	Familiarization phase	
	
	The first half of trials	The second half of trials
**Own-race-face condition**		
Mean	15.3	14.7
*SD*	1.05	1.12
**Other-race-face condition**		
Mean	15.3	14.7
*SD*	1.16	1.36

#### Test Trials

**FIGURE 5:**
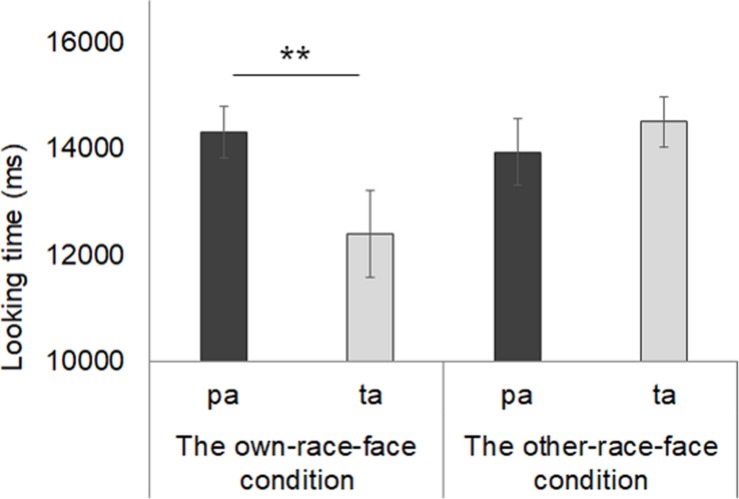
Mean total fixation times during the test phase in both the own-race-face condition and other-race-face condition. Dark gray bars represent the results for the novel trials (the presentation of auditory /pa/ with vegetable images) and light gray bars indicate the results for the familiarized trails (the presentation of auditory /ta/ with vegetable images). The error bars represent ±1 standard error of the mean. Asterisks indicate the significance level of the statistical differences: *p < 0.05, ** *p* < 0.01.

Mean total fixation times during the test phase in both the own-race-face condition and other-race-face condition are shown in Figure 5. In the own-race-face condition, the mean total looking time during the novel trial was 14.3 s (*SD* = 1.35, *SE* = 0.36), while that during the familiarized trial was 12.4 s (*SD* = 3.06, *SE* = 0.82). In the other-race-face condition, the mean total looking time during the novel trial was 13.94 s (*SD* = 2.33, *SE* = 0.62), while that during the familiarized trial was 14.52 s (*SD* = 1.76, *SE* = 0.47). A mixed ANOVA, with trial as a within-participants factor and the stimulus condition as a between-participants factor, showed no difference in total looking time across the test phase between the own-race-face condition and the other-race-face condition [*F*(1,26) = 1.54, *p* = 0.23, η^2^ = 0.03]. The ANOVA revealed a significant interaction between trial and stimulus condition [*F*(1,26) = 6.06, *p* = 0.02, η^2^ = 0.12].

Further, we conducted multiple *t-*tests (corrected using the Holm method) to compare the mean total looking time between the familiarized and novel trials in both race-face conditions. In the own-race-face condition, we found that infants showed a significant preference for the novel trial [*t*(13) = 3.14, *p* < 0.01]. However, no significant preference was found in the other-race-face condition [*t*(13) = 0.72, *p* = 0.49]. These results indicate that the infants perceived the McGurk effect (/ta/) during the familiarization phase in the own-race-face condition, but not in the other-race-face condition.

## Discussion

To support the results of fNIRS in Experiment 1, we used a familiarization/novelty preference method to confirm whether the infants could perceive the McGurk effect in the own-race-face condition and not that of the other-race-face. Our familiarization paradigm assumed a nested structure; (1) infants integrate audiovisual speech then perceive the syllable (the McGurk effect); (2) as a result, the infants are familiarized with the McGurk percept (/ta/) during the familiarization phase. If the infants perceive the McGurk effect, infants would show novelty preference toward the auditory syllable (/pa/) in the subsequent test phase. In our results, such novelty preference, which was found only in the own-race-face condition, indicates that 8- to 9-month-olds infants can perceive the McGurk effect. The behavioral data strongly support the difference in the brain responses to the McGurk effect between the two race stimuli in our fNIRS experiment, indicating that the McGurk stimulus by the own-race-face speaker evoked a significant activation in the infants’ brains.

## General Discussion

In the current study, we focused on infants’ brain responses to the McGurk effect to examine the difference in activation patterns between own-race-face and other-race-face conditions. In Experiment 1, we used fNIRS to find the presence of a mapping of the McGurk effect in the left temporal region and examine the difference in the activation patterns between own-race-face and other-race-face stimuli. The results from our fNIRS experiment indicated that (1) the McGurk stimuli induced changes in the concentrations of oxy-Hb in the bilateral temporal region, while the audiovisual-matched stimuli induced changes in the left temporal region; and that (2) a different activation pattern was found only in the own-race-face condition. These results were supported by the results of the behavioral experiment.

Our results showed the presence of a mapping of the McGurk effect in 8- to 9-month-old infants. That is, we found that the activation of bilateral temporal region was unique for the McGurk effect, which is different from the activation of the left temporal region when infants observed the audiovisual matched stimulus. However, the activation area for the McGurk effect was different from that in adults (Beauchamp et al., 2010; Nath and Beauchamp, 2012). We found that in infants, the bilateral temporal region was activated for the McGurk effect, while several studies showed that in adults, the left STS is responsible for the occurrence of the McGurk effect (e.g., Beauchamp et al., 2010; Nath and Beauchamp, 2012). This difference in the activation area between infants and adults may be due to the immature development of the McGurk effect in infants. Indeed, some studies suggest that the developmental trajectory of the McGurk effect continues until late childhood (e.g., McGurk and MacDonald, 1976; Sekiyama and Burnham, 2008), although it starts in infancy (Rosenblum et al., 1997; Burnham and Dodd, 2004; Desjardins and Werker, 2004). An fMRI study showed that the BOLD response to McGurk syllables in the left STS and bilateral fusiform gyri (areas of interests for fusiform face area) increased with the occurrence of McGurk effect in children who were less mature perceivers of the McGurk effect than adults (Nath et al., 2011). We consider that the activations of the bilateral temporal regions in our results came from infants who were immature perceivers of the McGurk effect. Whether and when a greater activation (supra-additive) of the left STS for processing audiovisual speech in infants is similar to that of adults remains to be discussed (e.g., Altvater-Mackensen and Grossmann, 2018). A future study should clarify this developmental process. We suspect that a similar brain pattern as that of adults would be observed in older children who have developed maturity in terms of the McGurk effect. A future study should examine the individual differences in this developmental process by testing the same infants with fNIRS and behavioral experiments.

Our results indicate that the other-race effect appears with the different activation patterns. The bilateral temporal region in infants was selectively activated to the McGurk effect when spoken by an own-race-face speaker and not by an other-race-face speaker. Different brain responses underlying perceptual narrowing have been reported in the development of face perception (e.g., the difference in brain responses between own- and other-race faces; Balas et al., 2011; Timeo et al., 2019) and speech perception (e.g., the difference in brain responses between native and non-native speech; Kuhl et al., 2014). Our findings addressed the different brain responses to the other-race effect in the context of the McGurk effect. The behavioral data from the present study also provide evidence that supports the difference in the brain response to the McGurk effect between the own-race-face and other-race-face conditions. By using the familiarization paradigm, the results of Experiment 2 showed that the infants perceived the McGurk effect when presented with the own-race-face stimulus and not the other-race-face stimulus. These results indicate that older infants can perceive the McGurk effect regardless of the syllables (Rosenblum et al., 1997; Burnham and Dodd, 2004; Desjardins and Werker, 2004); however, they may be limited to own-race speech. Nevertheless, the developmental process of the McGurk effect is protracted until late childhood (e.g., McGurk and MacDonald, 1976; Sekiyama and Burnham, 2008).

Our findings suggest the important role of experiences with own-race faces in the development of audiovisual speech perception. Our results showed that 8-to 9-month-old infants can perceive the McGurk effect only when the voice is paired with an own-race face that is familiar to the infants. This may imply that increased visual experiences with own-race faces make infants’ perceptual system tune to integrate a voice with an own-race face, with which the infants have more experience. This assumption also implies a possibility of the presence of perceptual narrowing in multisensory development (e.g., Lewkowicz and Ghazanfar, 2006; Pons et al., 2009). If the narrowing process underlie our results, then there would be developmental changes in the McGurk effect during the first year of life; that is, younger infants can perceive the McGurk effect regardless of speaker’s races, but older infants cannot perceive the McGurk effect when an other-race face is paired with a voice. A future study needs to clarify whether and how narrowing process emerges in the development of McGurk effect, by collecting data across multiple age groups as well as multiple ethnic groups.

Several factors can also lead to variances in the McGurk effect across participants. Previous studies have reported that in adults, the amount of the McGurk effect differed within (e.g., Nath and Beauchamp, 2012; Gurler et al., 2015) and between populations (Sekiyama and Tohkura, 1991; Sekiyama and Burnham, 2008). For instance, cultural difference factors into individual differences in the McGurk effect. Sekiyama and Tohkura (1991) reported that the amount of the McGurk effect was smaller in Japanese speakers than English speakers. The cultural factor may have influenced our findings. However, it is more important to note that a clear difference was found in the McGurk effect between the own-race-face and other-race-face conditions, even in the Japanese sample, who are considered to be weaker perceivers of the McGurk effect (Sekiyama and Tohkura, 1991). Sekiyama and Tohkura (1991) explained the cultural difference in the McGurk effect in terms of the different structures of the phonological systems of Japanese and English speakers. These results may imply that infants can perceive the McGurk effect from an own-race speaker; however, their perception is gradually modulated by the effect of the language structure after the age of acquiring a native language.

## Conclusion

In summary, the current study provides the first evidence for different brain responses, implying an other-race effect on the McGurk effect in 8- to 9-month-old infants. Our findings would support the hypothesis that perceptual narrowing is a modality-general, pan-sensory process (e.g., Lewkowicz and Ghazanfar, 2009).

## Data Availability Statement

The datasets generated for this study are available on request to the corresponding author.

## Ethics Statement

This experiment was conducted according to the Declaration of Helsinki and was approved by the Ethical Committee of Chuo University. Parents gave prior written informed consent for their children’s participation and for publication.

## Author Contributions

YU, SK, and MY contributed to the study design. Testing, data collection, and data analysis were performed by YU under the supervision of SK and MY. YU, SK, and MY performed the data interpretation. YU drafted the manuscript. SK and MY provided critical revisions. All authors approved the final version of the manuscript for submission.

## Conflict of Interest

The authors declare that the research was conducted in the absence of any commercial or financial relationships that could be construed as a potential conflict of interest.

## References

[B1] Altvater-Mackensen N. Grossmann T. (2018). Modality-independent recruitment of inferior frontal cortex during speech processing in human infants. *Dev. Cogn. Neurosci.* 34 130–138. 10.1016/j.dcn.2018.10.002 30391756 PMC6969291

[B2] Balas B. Westerlund A. Hung K. Nelson C. A. (2011). Shape, color and the other-race effect in the infant brain. *Dev. Sci.* 14 892–900. 10.1111/j.1467-7687.2011.01039.x 21676108 PMC4422220

[B3] Beauchamp M. S. Argall B. D. Bodurka J. Duyn J. H. Martin A. (2004). Unraveling multisensory integration: patchy organization within human STS multisensory cortex. *Nat. Neurosci.* 7 1190–1192. 10.1038/nn1333 15475952

[B4] Beauchamp M. S. Nath A. R. Pasalar S. (2010). fMRI-guided transcranial magnetic stimulation reveals that the superior temporal sulcus is a cortical locus of the mcgurk effect. *J. Neurosci.*, 30 2414–2417. 10.1523/JNEUROSCI.4865-09.2010 20164324 PMC2844713

[B5] Benavides-Varela S. Gervain J. (2017). Learning word order at birth: a NIRS study. *Dev. Cogn. Neurosci.* 25 198–208. 10.1016/j.dcn.2017.03.003 28351534 PMC6987835

[B6] Burnham D. Dodd B. (2004). Auditory-visual speech integration by prelinguistic infants: perception of an emergent consonant in the McGurk effect. *Dev. Psychobiol.* 45 204–220. 10.1002/dev.20032 15549685

[B7] Calvert G. A. Campbell R. Brammer M. J. (2000). Evidence from functional magnetic resonance imaging of crossmodal binding in the human heteromodal cortex. *Curr. Biol.* 10 649–657. 10.1016/s0960-9822(00)00513-3 10837246

[B8] Desjardins R. N. Werker J. F. (2004). Is the integration of heard and seen speech mandatory for infants? *Dev. Psychobiol.* 45 187–203. 10.1002/dev.20033 15549681

[B9] Eimas P. D. Siqueland E. R. Jusczyk P. Vigorito J. (1971). Speech perception in infants. *Science* 171 303–306. 5538846 10.1126/science.171.3968.303

[B10] Gurler D. Doyle N. Walker E. Magnotti J. Beauchamp M. (2015). A link between individual differences in multisensory speech perception and eye movements. *Attent. Percept. Psychophys.* 77 1333–1341. 10.3758/s13414-014-0821-1 25810157 PMC4437244

[B11] Hannon E. E. Trehub S. E. (2005a). Metrical categories in infancy and adulthood. *Psychol. Sci.* 16 48–55. 10.1111/j.0956-7976.2005.00779.x 15660851

[B12] Hannon E. E. Trehub S. E. (2005b). Tuning in to musical rhythms: infants learn more readily than adults. *Proc. Natl. Acad. Sci. U.S.A.* 102 12639–12643. 10.1073/pnas.0504254102 16105946 PMC1194930

[B13] Issard C. Gervain J. (2017). Adult-like processing of time-compressed speech by newborns: a NIRS study. *Dev. Cogn. Neurosci.* 25 176–184. 10.1016/j.dcn.2016.10.006 27852514 PMC6987815

[B14] Jusczyk P. W. Copan H. Thompson E. (1978). Perception by 2-month-old infants of glide contrasts in multisyllabic utterances. *Percept. Psychophys.* 24 515–520. 10.3758/bf03198777 750994

[B15] Kelly D. J. Liu S. Lee K. Quinn P. C. Pascalis O. Slater A. M. (2009). Development of the other-race effect during infancy: evidence toward universality? *J. Exp. Child Psychol.* 104 105–114. 10.1016/j.jecp.2009.01.006 19269649 PMC3740564

[B16] Kelly D. J. Quinn P. C. Slater A. M. Lee K. Ge L. Pascalis O. (2007). The other-race effect develops during infancy: evidence of perceptual narrowing. *Psychol. Sci.* 18 1084–1089. 10.1111/j.1467-9280.2007.02029.x 18031416 PMC2566514

[B17] Kobayashi M. Macchi Cassia V. Kanazawa S. Yamaguchi M. K. Kakigi R. (2018). Perceptual narrowing towards adult faces is a cross-cultural phenomenon in infancy: a behavioral and near-infrared spectroscopy study with Japanese infants. *Dev. Sci.* 21:e12498. 10.1111/desc.12498 27921339 PMC5763342

[B18] Krasotkina A. Götz A. Höhle B. Schwarzer G. (2018). Perceptual narrowing in speech and face recognition: evidence for intra-individual cross-domain relations. *Front. Psychol.* 9:1711. 10.3389/fpsyg.2018.01711 30258388 PMC6144632

[B19] Kuhl P. K. Meltzoff A. N. (1982). The bimodal perception of speech in infancy. *Science* 218 1138–1141. 10.1126/science.7146899 7146899

[B20] Kuhl P. K. Meltzoff A. N. (1984). The intermodal representation of speech in infants. *Infant Behav. Dev.* 7 361–381.

[B21] Kuhl P. K. Ramírez R. R. Bosseler A. Lin J. L. Imada T. (2014). Infants’ brain responses to speech suggest analysis by synthesis. *Proc. Natl. Acad. Sci. U.S.A.* 111 11238–11245. 10.1073/pnas.1410963111 25024207 PMC4128155

[B22] Kushnerenko E. Teinonen T. Volein A. Csibra G. (2008). Electrophysiological evidence of illusory audiovisual speech percept in human infants. *Proc. Natl. Acad. Sci. U.S.A.* 105 11442–11445. 10.1073/pnas.0804275105 18682564 PMC2516214

[B23] Lewkowicz D. J. Ghazanfar A. A. (2006). The decline of cross-species intersensory perception in human infants. *Proc. Natl. Acad. Sci. U.S.A.* 103 6771–6774. 10.1016/j.brainres.2008.03.084 16618919 PMC1458955

[B24] Lewkowicz D. J. Ghazanfar A. A. (2009). The emergence of multisensory systems through perceptual narrowing. *Trends Cogn. Sci.* 13 470–478. 10.1016/j.tics.2009.08.004 19748305

[B25] Lewkowicz D. J. Sowinski R. Place S. (2008). The decline of cross-species intersensory perception in human infants: underlying mechanisms and its developmental persistence. *Brain Res.* 1242 291–302. 10.1016/j.brainres.2008.03.084 18486112 PMC2612707

[B26] Lloyd-Fox S. Richards J. E. Blasi A. Murphy D. G. M. Elwell C. E. Johnson M. H. (2014). Co-registering fNIRS with underlying cortical areas in infants. *Neurophotonics* 1:025006. 10.1117/1.NPh.1.2.025006 25558463 PMC4280679

[B27] Maris E. Oostenveld R. (2007). Nonparametric statistical testing of EEG-andMEG-data. *J. Neurosci. Methods* 164 177–190. 10.1016/j.jneumeth.2007.03.024 17517438

[B28] Massaro D. W. Thompson L. A. Barron B. Laren E. (1986). Developmental changes in visual and auditory contributions to speech perception. *J. Exp. Child Psychol.* 41 93–113. 10.1016/0022-0965(86)90053-6 3950540

[B29] McGurk H. MacDonald J. W. (1976). Hearing lips and seeing voices. *Nature* 264 746–748. 1012311 10.1038/264746a0

[B30] Nath A. R. Beauchamp M. S. (2012). A neural basis for interindividual differences in the mcgurk effect, a multisensory speech illusion. *Neuroimage* 59 781–787. 10.1016/j.neuroimage.2011.07.024 21787869 PMC3196040

[B31] Nath A. R. Fava E. E. Beauchamp M. S. (2011). Neural correlates of interindividual differences in children’s audiovisual speech perception. *J. Neurosci.* 31 13963–13971. 10.1523/JNEUROSCI.2605-11.2011 21957257 PMC3203203

[B32] Otsuka Y. Nakato E. Kanazawa S. Yamaguchi M. K. Watanabe S. Kakigi R. (2007). Neural activation to upright and inverted faces in infants measured by near infrared spectroscopy. *Neuroimage* 34 399–406. 10.1016/j.neuroimage.2006.08.013 16997577

[B33] Pascalis O. de Haan M. Nelson C. A. (2002). Is face processing species-specific during the first year of life? *Science* 296 1321–1323. 10.1126/science.1070223 12016317

[B34] Pascalis O. Loevenbruck H. Kandel S. Tanaka J. M. Lee K. (2014). On the links among face processing, language processing, and narrowing during development. *Child Dev. Perspect.* 8 65–70. 10.1111/cdep.12064 25254069 PMC4164271

[B35] Patterson M. L. Werker J. F. (1999). Matching phonetic information in lips and voice is robust in 4.5-month-old infants. *Infant Behav. Dev.* 22 237–247. 10.1016/j.cognition.2008.05.009 18590910

[B36] Patterson M. L. Werker J. F. (2002). Infants’ ability to match dynamic phonetic and gender information in the face and voice. *J. Exp. Child Psychol.* 81 93–115. 10.1006/jecp.2001.2644 11741376

[B37] Piazza E. A. Iordan M. C. Lew-Williams C. (2017). Mothers consistently alter their unique vocal fingerprints when communicating with infants. *Curr. Biol.* 27 3162–3167. 10.1016/j.cub.2017.08.074 29033333 PMC5656453

[B38] Pons F. Lewkowicz D. J. Soto-Faraco S. Sebastián-Gallés N. (2009). Narrowing of intersensory speech perception in infancy. *Proc. Natl. Acad. Sci. U.S.A.* 106 10598–10602. 10.1073/pnas.0904134106 19541648 PMC2705579

[B39] Rosenblum L. D. Schmuckler M. A. Johnson J. A. (1997). The McGurk effect in infants. *Percept. Psychophys.* 59 347–357. 9136265 10.3758/bf03211902

[B40] Sato K. Kanazawa S. Yamaguchi M. K. (2017). Infants’ perception of lightness changes related to cast shadows. *PLoS One* 12:e0173591. 10.1371/journal.pone.0173591 28296912 PMC5351879

[B41] Sekiyama K. Burnham D. (2008). Impact of language on development of auditory-visual speech perception. *Dev. Sci.* 11 303–317. 10.1111/j.1467-7687.2008.00677.x 18333984

[B42] Sekiyama K. Tohkura Y. (1991). McGurk effect in non-English listeners: few visual effects for Japanese subjects hearing Japanese syllables of high auditory intelligibility. *J. Acoust. Soc. Am.* 90 1797–1805. 10.1121/1.401660 1960275

[B43] Timeo S. Brigadoi S. Farroni T. (2019). Perception of Caucasian and African faces in 5- to 9-month-old Caucasian infants: a functional near-infrared spectroscopy study. *Neuropsychologia* 126 3–9. 10.1016/j.neuropsychologia.2017.09.011 28916447

[B44] Ujiie Y. Asai T. Tanaka A. Wakabayashi A. (2015). The McGurk effect and autistic traits: an analogue perspective. *Lett. Evol. Behav. Sci.* 6 9–12.

[B45] Ujiie Y. Asai T. Wakabayashi A. (2018a). Individual differences and the effect of face configuration contexts in the McGurk effect. *Exp. Brain Res.* 236 973–984. 10.1007/s00221-018-5188-4 29383400

[B46] Ujiie Y. Yamashita W. Fujisaki W. Kanazawa S. Yamaguchi M. K. (2018b). Crossmodal association of auditory and visual material properties in infants. *Sci. Rep.* 8:9301. 10.1038/s41598-018-27153-2 29915205 PMC6006328

[B47] Werker J. F. Tees R. C. (1983). Developmental changes across childhood in the perception of non-native speech sounds. *Can. J. Psychol.* 37 278–286. 10.1037/h0080725 6616342

[B48] Werker J. F. Tees R. C. (2002). Cross-language speech perception: evidence for perceptual reorganization during the first year of life. *Infant Behav. Dev.* 25 121–133. 10.1016/j.cognition.2007.07.002 17707789

[B49] Xiao N. G. Mukaida M. Quinn P. C. Pascalis O. Lee K. Itakura S. (2018). Narrowing in face and speech perception in infancy: developmental change in the relations between domains. *J. Exp. Child Psychol.* 176 113–127. 10.1016/j.jecp.2018.06.007 30149243

[B50] Yang J. Kanazawa S. Yamaguchi M. K. Kuriki I. (2016). Cortical response to categorical color perception in infants investigated by near-infrared spectroscopy. *Proc. Natl. Acad. Sci. U.S.A.* 113 2370–2375. 10.1073/pnas.1512044113 26858441 PMC4780595

