# Supplementary material for: The Other-Race-Effect on Audiovisual Speech Integration in Infants: A NIRS Study
Source: Front Psychol. 2020 May 15;11:971. doi: 10.3389/fpsyg.2020.00971 (PMC7243679; doi:10.3389/fpsyg.2020.00971)
Supplement: Supplementary file 1 [file Data_Sheet_1.pdf]

Supprementay materials

Results for experiment 1

The time course of the average changes in total-Hb concentration in all conditions were shown in figure S1.

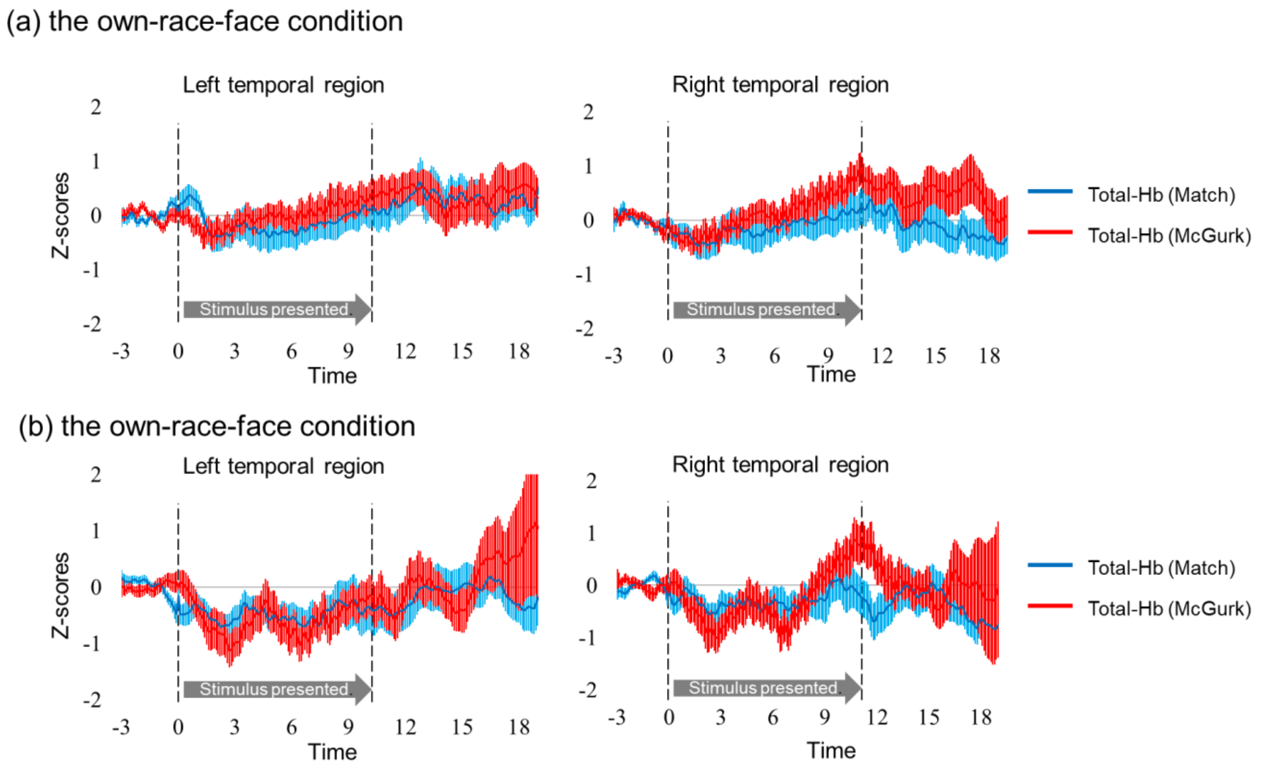

Figure S1. Time course of the changes in the total hemoglobin (total-Hb) concentrations. Total-Hb concentrations were averaged in all groups during each condition in the left and right temporal regions; (a) the results for the own-race-face condition, and (b) the results for the other-race-face condition. Blue lines and red lines represent the mean Z-score during the matched and mismatched trials, respectively. The vertical dashed lines at 0 and 9.6 s indicate the onset and offset of the test stimulus presentation, respectively.

Results of the ANOVA in Experiment 1

We performed a further statistical analysis of mean *Z*-scores during the 12–16 s after stimulus onset in order to compare the concentrations of oxy-Hb (and deoxy-Hb) between the two race-face conditions. We conducted a mixed ANOVA with hemisphere (the right and left temporal regions) and stimulus condition (match and McGurk) as within-participants factors and the speaker's race (the own-race-face and the other-race-face) as a between-participants factor. For the concentration of oxy-Hb, the ANOVA showed that the main effect of the speaker's race was marginally significant (F[1,32] = 3.36, p = .076, η^2 = .10), while the main effects of hemisphere (F[1,32] = .04, p = .84, η^2 = .0001) and stimulus condition (F[1,32] = .47, p = .50, η^2 = .005) were not significant. There were no significant

interactions (e.g., the speaker's race × hemisphere × stimulus condition, $F[1,32] = .02$, $p = .89$, $\eta^2 = .00001$). In the concentration of deoxy-Hb, there were no significant main effects ($F[1,32] = .39$, $p = .54$, $\eta^2 = .006$; $F[1,32] = .60$, $p = .44$, $\eta^2 = .001$; and $F[1,32] = .85$, $p = .37$, $\eta^2 = .009$). There were no significant interactions (e.g., the speaker's race × hemisphere × stimulus condition, $F[1,32] = .15$, $p = .70$, $\eta^2 = .0004$). These results indicate that the changes in the concentration of oxy-Hb in the temporal brain regions were higher when the infants observed audiovisual speech stimuli under the own-race face condition than the other-race face condition. In contrast, changes in the concentration of deoxy-Hb did not reach significance in the temporal brain regions, as was shown by the results of *t*-tests.

fNIRS experiment in control condition

To confirm whether infant brain activity in the left temporal region was found when they can integrate auditory and visual speech, we conducted a control experiment by measuring another 10 infants' NIRS responses in bilateral temporal regions. We used audiovisual matched (auditory /a/ and visual /a/) and mismatched (auditory /a/ and visual /i/) stimuli, both of which were created using the own-race speaker's speech. We presented the infants with audiovisual matched trials and audiovisual mismatched trials alternately, after presenting baseline trials. We hypothesized that the left temporal region in infants would be activated when the infants observe audiovisual matched speech, but not when they observe audiovisual mismatched speech.

Methods

Participants

All infants were full term at birth and were healthy at the time of the experiments. The participants were 10 healthy Japanese infants aged 8- to 9-month-old (six girls and four boys; mean age = 247.5 days, range = 226–277 days). Ethical approval for this study was obtained from the local Ethical Committee. Written informed consent was obtained from the parents of the participants.

Stimuli and procedure

We used audiovisual speech stimuli of recordings of a woman's IDS for two vowels (/a/ and /i/). The speaker was a Japanese East-Asian woman (22 years old). The visual stimuli (800 × 450 pixels) and voice were recorded in the same setting as the syllable recording. We created matched stimuli (auditory /a/ and visual /a/) and mismatched stimuli (auditory /a/ and visual /i/) using Adobe Premiere Pro CS6 (Adobe Systems). The congruency of the stimuli was based on the auditory information. The duration of each stimulus was 3.2 s. The sequence of the stimulus presentation consisted of two test trials and a baseline trial. The matched and mismatched stimuli were use in the test trials. The matched and mismatched stimuli were presented three times each, in alternating trials. The duration of the test trial was 9.6 s. In a baseline period, dynamic random dot patterns (800 × 450 pixels) with an auditory white noise were displayed repetitively once every 3.2 s, of duration of at least

9.6 s.

In this experiment, the procedure, apparatus and data analysis were identical to those in experiment 1.

Results

Hemodynamic data were obtained from 10 infants who had more than three valid trials for each test trial. As in experiment 1, we normalized the raw data of the hemodynamic response using the mean and standard deviation (SD) of the baseline period for each channel and each participant before applying statistical analyses because the raw data could not be averaged directly across participants and channels. Subsequently, we averaged the Z-scores of oxygenated hemoglobin (oxy-Hb) and deoxygenated hemoglobin (deoxy-Hb) across 12 channels in each hemisphere and compared them to the baseline. Figure S2 shows the time course of the average changes in concentrations of oxy-Hb and deoxy-Hb during the presentation of matched and mismatched trials

We performed statistical analyses on the mean Z-scores for a time window from 12 to 16 s after stimulus onset (Figure S3). In the left temporal region, the concentration of oxy-Hb increased significantly during the matched trials (t [9] = 2.98, p = .02) but not during the mismatched trials (t [9] = 1.00, p = .34). In contrast, there was no significant difference in the concentration of oxy-Hb in the right temporal region during the matched trials (t [9] = 1.70, p = .12) or mismatched trials (t [9] = .42, p = .68). This result indicates that infant brain activity in the left temporal region may reflect audiovisual integration of auditory and visual speech.

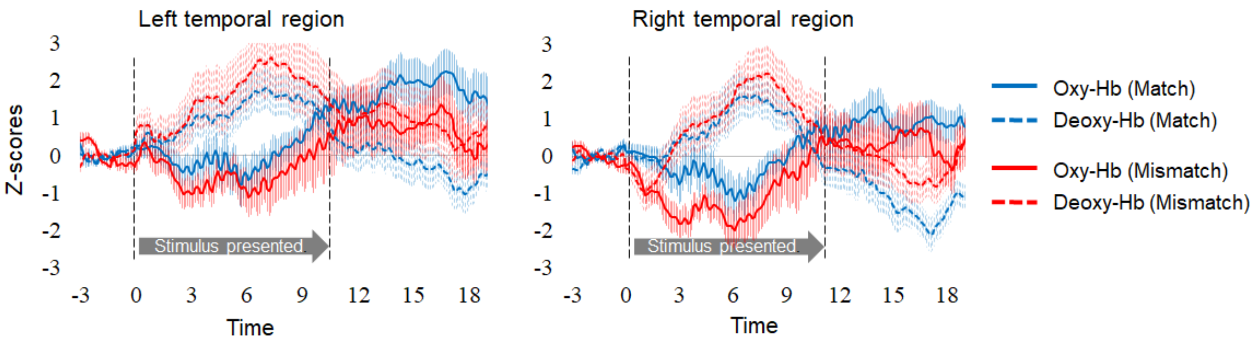

Figure S2. Time course of the changes in the oxygenated hemoglobin (Oxy-Hb) and deoxygenated hemoglobin (Deoxy-Hb) concentrations. Oxy-Hb and Deoxy-Hb concentrations averaged in the left and right temporal regions. Solid lines represent the change in Oxy-Hb, and dotted lines represent the change in Deoxy-Hb. Blue lines and red lines represent the mean Z-score during the matched and mismatched trials, respectively. The vertical dashed lines at 0 and 9.6 s indicate the onset and offset of the test stimulus presentation, respectively.

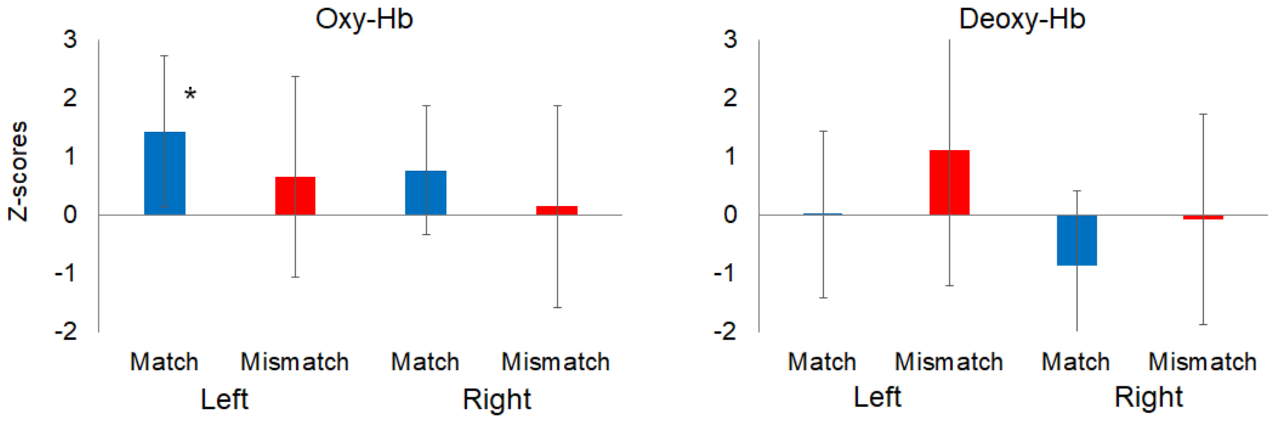

Figure S3. Mean Z-scores of oxygenated hemoglobin (Oxy-Hb) and deoxygenated hemoglobin (Deoxy-Hb) in the left temporal (Left) and right temporal (Right) regions. The Oxy-Hb results are shown in the left panel and the Deoxy-Hb results are shown in the right panel. Each bar represents the mean Z-score of Oxy-Hb (or Deoxy-Hb) averaged across 12–16 s after stimulus onset. Blue bars and red bars represent the results for the matched and the mismatched conditions, respectively. The error bars represent 95% confidence interval of the mean. Asterisks indicate the significance level of the statistical differences against baseline (0): $*p < .05$.
